# Different Targeting Ligands-Mediated Drug Delivery Systems for Tumor Therapy

**DOI:** 10.3390/pharmaceutics16020248

**Published:** 2024-02-07

**Authors:** Shuxin Yan, Jintong Na, Xiyu Liu, Pan Wu

**Affiliations:** 1 State Key Laboratory of Targeting Oncology, National Center for International Research of Bio-Targeting Theranostics, Guangxi Key Laboratory of Bio-Targeting Theranostics, Collaborative Innovation Center for Targeting Tumor Diagnosis and Therapy, Guangxi Medical University, Nanning 530021, China; yanshuxin@sr.gxmu.edu.cn (S.Y.); najintong@sr.gxmu.edu.cn (J.N.); 2 School of Pharmacy, Guangxi Medical University, Nanning 530021, China

**Keywords:** targeted drug delivery system, targeting ligand, folic acid, carbohydrates, peptides, aptamers, antibodies, tumor therapy

## Abstract

Traditional tumor treatments have the drawback of harming both tumor cells and normal cells, leading to significant systemic toxic side effects. As a result, there is a pressing need for targeted drug delivery methods that can specifically target cells or tissues. Currently, researchers have made significant progress in developing targeted drug delivery systems for tumor therapy using various targeting ligands. This review aims to summarize recent advancements in targeted drug delivery systems for tumor therapy, focusing on different targeting ligands such as folic acid, carbohydrates, peptides, aptamers, and antibodies. The review also discusses the advantages, challenges, and future prospects of these targeted drug delivery systems.

## 1. Introduction

Cancer is a chronic disease that presents a significant risk to human life. In 2020, the global incidence of new cancer cases was estimated to have reached 19.3 million, leading to approximately 10 million fatalities. This alarming statistic represents about one-sixth of all global deaths [1,2]. To combat this life-threatening disease, numerous cancer treatments have been discovered, including surgery, chemotherapy, immunotherapy, radiation therapy, phototherapy, thermotherapy, and more. Alongside these treatments, various drugs have been developed, such as small-molecule chemotherapeutic drugs and nanoparticles (NPs). However, it has been observed that these drugs do not effectively differentiate between pathological and normal cells, leading to toxicities [3,4]. Therefore, there is an urgent need for new cancer therapies that are targeted and have reduced toxicity [5]. In recent decades, targeted therapies have emerged as a promising approach to address the issues of high cytotoxicity and lack of tumor specificity in alternative cancer treatments [6].

Targeting ligands, such as folic acid [7], carbohydrates [8], peptides [9], aptamers [10], and antibodies [11], exhibit structural diversity and possess unique strengths and weaknesses. They play a crucial role in distinguishing between pathological and normal tissues. These ligands specifically bind to receptors expressed on tumor cells, minimizing the damage caused by cytotoxic drugs to normal cells. While each type of targeted ligand is well-documented in the references provided, Table 1 offers a concise overview of each group for easy reference. Despite their diverse targets and entry mechanisms into tumor cells, these targeting ligands have demonstrated promising results in anticancer therapeutic studies. By functionalizing small molecule drugs and nanoparticles that recognize targeting ligands in cancer cells, a targeted drug delivery system can be created. This system enables selective delivery of drugs to cancer cells, significantly enhancing therapeutic efficacy while reducing toxicity [12]. 

**Figure 1:**
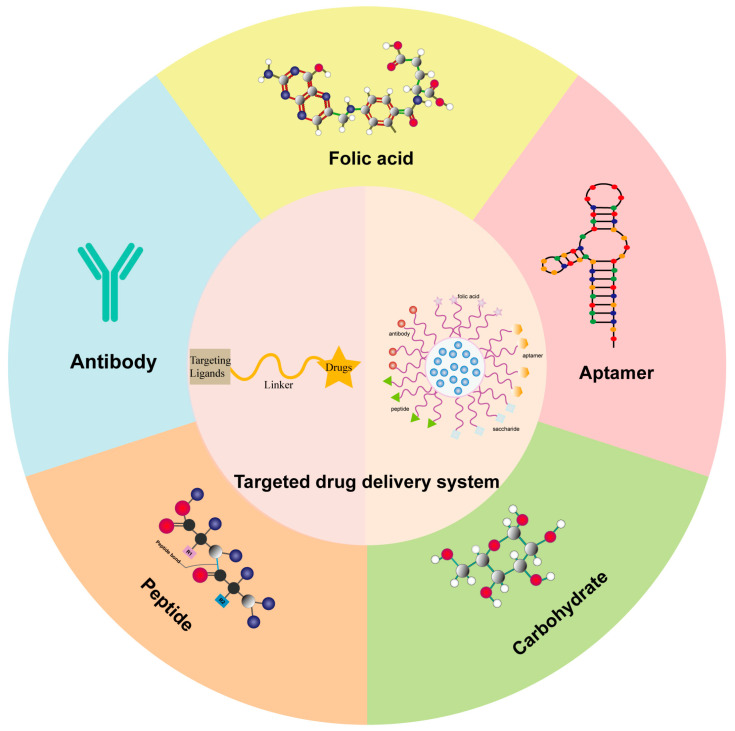
Different targeting ligands-mediated drug delivery systems.

The targeted drug delivery system is both simple and efficient. In recent years, several targeting ligands, including folic acid, carbohydrates, peptides, aptamers, and antibodies, have been utilized in targeted drug delivery systems [13] (Figure 1). These targeting ligands accurately recognize and specifically bind to markers expressed on targeted tumor cells. This recognition allows small molecule drugs that directly bind to the targeting ligand or active drugs carried by targeting ligand-conjugated nanocarriers to be delivered exclusively to cells expressing the appropriate receptor [14]. As a result, normal cells are not affected by the targeting carriers. The successful use of these targeted drug delivery systems has effectively enhanced the therapeutic efficacy of cytotoxic drugs and reduced their toxic side effects. This innovation is being actively developed globally and is expected to revolutionize current cancer treatment strategies. 

This review focuses on the research progress of targeted drug delivery systems based on different targeting ligand-mediated delivery systems, including folic acid, carbohydrates, peptides, aptamers, and antibodies. Additionally, we discuss the current challenges and future prospects of these different targeting ligand-mediated drug delivery systems.

## 2. Folate-Mediated Targeted Drug Delivery System for Tumor Treatment

**Figure 2:**
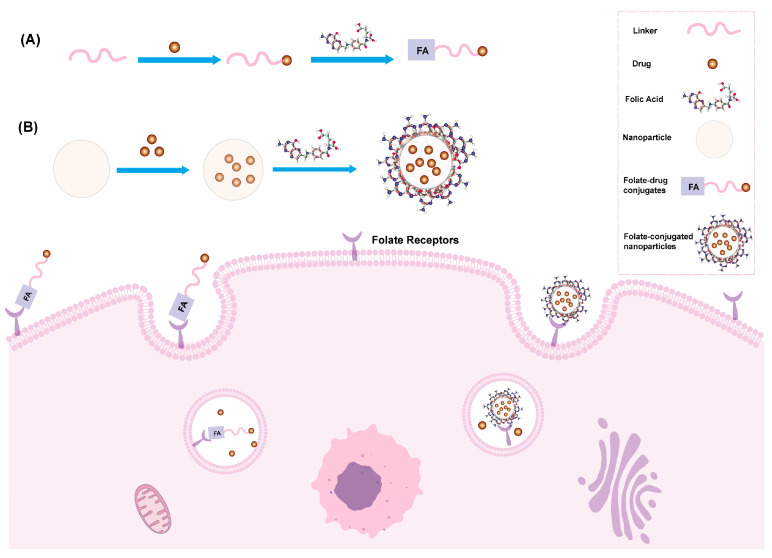
Folate-mediated targeted drug delivery system. (**A**) Compositional process of folate-drug conjugates. (**B**) Compositional process of folate-conjugated nanoparticles.

Folic acid (FA) is a small-molecule vitamin that plays a crucial role in the biosynthesis of nucleotide bases and cell proliferation. It is transported through receptor-mediated endocytosis using cell membrane-associated proteins or the folate receptor (FR) [15]. While the folate receptor is expressed at low levels in normal tissues, it is overexpressed in a variety of cancer types, including breast, ovarian, endometrial, renal, lung, head and neck, brain, colon, and medullary cancers. Because of its small size, chemical simplicity and lack of immunogenicity, folic acid is widely used as a tumor-targeting ligands for precise delivery of therapeutic agents to diseased cells or tissues [16]. Currently, folate-targeted ligand-guided drug delivery systems involve two different pathways: one involves directly conjugating folic acid to small-molecule drugs to form folate drug-conjugates, while the other involves combining folic acid with nanomaterials to form folate-conjugated nanoparticles [17,18] (Figure 2).

### 2.1. Folate-Drug Conjugates

Due to the superior tumor-targeting capabilities of folic acid over small molecule chemotherapeutic drugs, researchers created folate-drug conjugates through the covalent conjugating of the targeting molecule folic acid and the small molecule chemotherapeutic drugs. These conjugates were then used to deliver the small molecule drugs to tumor cells that expressed the folate-receptor (FR), thereby increasing the therapeutic efficacy of these chemotherapeutic drugs [19].

In folate-drug conjugates, the amino group (NH2) on pteridine folate can be covalently linked to the small molecule drug through chemical bonds such as acylhydrazone, amide, and ester groups. In a study, researchers modified folic acid on dopamine, which is used as a catechol linker (Cat), and then coupled it to the boronic acid of Bortezomib (BTZ) via a boronic acid ester bond to develop a novel pH-sensitive FA-modified BTZ coupler (FA-Cat-BTZ) for cancer-specific drug delivery and therapy. The results showed that the FA-Cat-BTZ formulation exhibited significantly enhanced proteasome inhibition and induction of apoptosis. This improvement was attributed to FR-mediated endocytosis and the rapid release of the drug triggered by intracellular pH changes. Comparative analysis revealed that FA-Cat-BTZ outperformed BTZ, BTZ-mannitol derivatives, and FA-PEG-Cat-BTZ in terms of FR+ cellular uptake, permeation, and anticancer activity. These findings suggest that the FA-Cat-BTZ coupling exhibits broad promise as a tumor-targeted proteasome inhibitor for achieving specific drug delivery to tumors and improving the efficiency of cancer therapy [17]. Based on the promising antitumor activity and low toxicity in preclinical models, researchers have taken folate drug conjugates through clinical trials to further evaluate their therapeutic efficacy and safety in humans. One such conjugate, EC145, which targets desacetylvinblastine hydrazide, has entered phase 2 trials for various types of cancers. Another conjugate, EC0489, has been developed with a peptidoglycan spacer to reduce liver toxicity, and is currently being investigated in phase 1 trials for metastatic solid tumors. Additionally, a folic acid-targeted double-bullet drug called EC0225 is being studied in a phase 1 clinical trial for the same indications [20]. However, it has been observed that the use of folate drug conjugates in clinical trials is more challenging compared with preclinical models, as some patients may develop resistance to these conjugates, resulting in reduced treatment efficacy. Furthermore, folic acid drug conjugates may also cause adverse effects such as nausea and vomiting. Therefore, researchers need to continue exploring new folate drug conjugates for clinical trials to overcome these difficulties.

Compared with non-targeted therapies, the folate-drug conjugate system offers greater flexibility in terms of drug optimization, reduces the exposure of healthy cells to cytotoxic drugs, and minimizes adverse toxicity. Despite these achievements, the number of folate-drug conjugates is limited, and further evaluation of their efficacy is required. Additionally, the exact mechanism of action for these conjugates remains unclear. Therefore, future research should focus on continuous optimization of synthesis technology and further elucidation of the mechanism, enabling their application in a wider range of cancer therapies.

### 2.2. Folate-Conjugated Nanoparticles

Folic acid may be one of the suitable options for targeting tumor cells in nanoparticle-based cancer therapy [21]. This is due to the overexpression of folate receptors on the surface of some tumor cells, making folate targeting an effective strategy to improve the efficiency of cancer therapy. Therefore, the use of folate-conjugated nanoparticles to deliver multiple drugs to tumor tissues has attracted increasing attention.

Folic acid can be attached to nanoparticles through covalent and noncovalent coupling methods. Currently, researchers are using covalent bonds to attach folic acid to various nanomaterials such as antitumor drugs, liposomes, lactosomes, carbon nanotubes, dendrimers, and tumor-targeting polymers [22]. For instance, novel FA-PLGA nanoparticles loaded with oxaliplatin have been developed for the treatment of colorectal cancer cells. These nanoparticles have shown enhanced efficiency in regulating tumor progression, increasing apoptosis, reducing drug resistance, and improving cytotoxicity and cell death [23]. In breast cancer models, loading adriamycin into folate-coupled magnetic nanoparticles has been shown to significantly increase drug uptake by tumor cells, enhance drug accumulation in the body and inhibit tumor growth [24]. Folic acid-conjugated silk nanoparticles have also been used to target delivery of IB to cancer cell lines, taking advantage of the overexpression of folic acid receptors on their surfaces. This approach has improved the therapeutic effect of nanomedicines in tumor cells or tissues [25]. Additionally, folic acid can be physisorbed onto nanomaterial surfaces to achieve targeted drug delivery. In a study, researchers physisorbed carbon nanotubes loaded with raloxifene hydrochloride (RLX) onto folate ligands for targeted treatment of breast cancer. The results demonstrated that this approach significantly increased cellular uptake through folate-folate receptor interaction, thereby enhancing therapeutic efficacy [26].

Folate-conjugated nanoparticles have proven to be an effective strategy for anticancer therapy. They not only selectively target tumor cells and facilitate intracellular targeting, but also enhance drug utilization and improve the efficiency of cancer treatment [27]. Furthermore, the surface functionalization of nanoparticles with folic acid enhances their stability, biocompatibility, biodegradability, non-toxicity, loading capacity, potential for active tumor accumulation, ability to overcome drug resistance in cancer cells, and excellent pharmacokinetic parameters [28]. Despite these advancements, most existing studies on folate-conjugated nanoparticles have been limited to basic preclinical research, with clinical trials remaining unrealized. Therefore, further investigation and formulation of new folic acid-based drugs are warranted for future clinical exploration.

## 3. Carbohydrate-Mediated Targeted Drug Delivery System for Tumor Treatment

**Figure 3:**
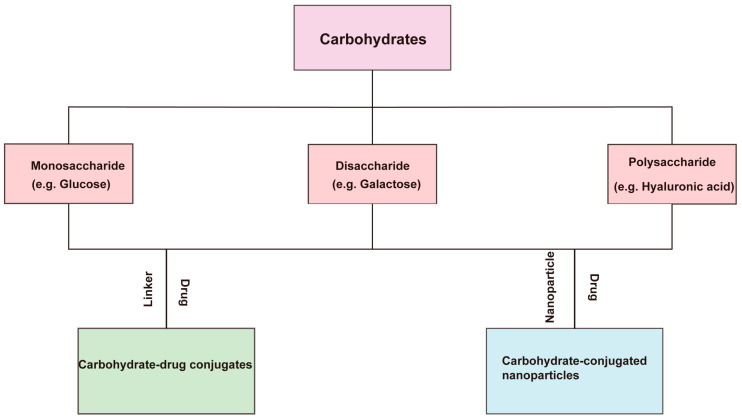
Carbohydrate-mediated targeted drug delivery system. It means that carbohydrates are categorized into three types: monosaccharides, disaccharides, and polysaccharides, and all three of these different structural carbohydrates can be used to form carbohydrate-drug conjugates with small molecule drugs or carbohydrate conjugated nanoparticles with nanoparticles.

Carbohydrates (or saccharides) are one of the widespread and abundant biomolecules in nature consisting of oxygen, carbon, and hydrogen. According to molecular size and chemical properties, they can be categorized into a number of different groups such as monosaccharides, disaccharides, and polysaccharides [29]. Some carbohydrates targeting specific receptors such as glucose, galactose, and hyaluronic acid have multiple reactive hydroxyl groups that can be readily introduced onto the surface of drug carriers by chemical modification [30]. The capacity of carbohydrates to bind to specific cell surface receptors and their biocompatibility make them attractive candidates as targeting ligands for applications involving tailored delivery of medicines and genes to particular cells. This section focuses on the application of carbohydrates as targeted ligands for drug delivery in tumor therapy [31,32] (Figure 3).

### 3.1. Carbohydrate-Drug Conjugates

It was known that cancerous cells exhibited an enhanced metabolism, greater need for glucose, and elevated uptake of glucose, resulting in an augmented expression of glucose transporter (GLUT) [33]. The intensified absorption of glucose or other saccharide by malignant cells implies that saccharide compounds with biologically active substances might present themselves as hopeful prospects for targeted medications. Thus, glycoconjugates hold the capacity to be exclusively assimilated by cancer cells, while causing minimal impact on normal cells [34].

Among carbohydrate-drug conjugates, Glycoconjugated prodrugs have been extensively studied in the field of anticancer drugs. These prodrugs typically consist of a known anticancer drug connected to a sugar unit through a glycosidic bond or various linkers such as esters, amides, ureas, and succinic acids [35]. One of the earliest glycoconjugates, Glufosfamide, was synthesized in 1995 by Wiessler et al. This groundbreaking design involved linking β-D-glucose to the alkylating moiety of ifosfamide, resulting in increased cancer-selective uptake of the conjugate mediated by GLUT [36]. Since then, researchers have developed various glycoconjugates targeted to GLUT or Asialoglycoprotein receptor (ASPGR), using cytotoxic molecules like chlorambucil, paclitaxel, and adriamycin [37]. For instance, Mao et al. reported the glucose conjugates of paclitaxel in 2018. Single or double glucose moieties were affixed to paclitaxel via succinate linkers. The resulting single glycosylated paclitaxel (GluSA-PTX) and double glycosylated paclitaxel (bis-GluSA-PTX) conjugates demonstrated potent cytotoxicity against breast cancer cells and enhanced water solubility in comparison to the original drug [38]. Furthermore, researchers successfully synthesized a novel conjugate of methotrexate and glucose known as Glu-MTX in 2021. The Glu-MTX compound was formed by connecting a sugar moiety through a glycosidic linkage and an MTX moiety through a carbamate linkage. Through activity assays, it was observed that the glycoconjugate MTX-Glu displayed potent cytotoxic effects in vitro against a diverse range of cancer cell lines, much like unaltered MTX. This finding was further corroborated by in vivo investigations conducted on mice with breast cancer. Of utmost significance, the MTX conjugate exhibited minimal toxicity towards noncancerous cells, thereby significantly enhancing the drug’s selectivity [31].

This type of glycoconjugate acts as a selective target for tumor tissues, reducing the toxicity of anticancer drugs to normal tissues and aiding in the treatment of cancer. The compound shows promise as a potential therapy due to the high glucose demand of cancer cells and the presence of GLUT or ASPGR receptors in tumor sites. However, further research is needed to fully understand the role of glucose as a targeting ligand, as most studies have been conducted in vitro. Additional correlation tests are necessary to confirm these findings.

### 3.2. Carbohydrate-Conjugated Nanoparticles

In recent decades, there has been increasing interest in utilizing carbohydrates as active targeting ligands on the surface of nanoparticles [8]. These carbohydrates, with their diverse chemical compositions and side chains, can be introduced onto NPs through non-covalent or covalent bonds. This approach has shown promise in improving the therapeutic efficacy of multifunctional nanocarriers by enhancing cellular uptake [39]. In carbohydrate-targeted nanodrug delivery systems, specific carbohydrates such as galactose, lactate, and hyaluronic acid can function as targeting ligands that are recognized by corresponding receptors like ASGPR and CD44. It is possible to target particular organs, tissues, and cells thanks to ligand-receptor-mediated active targeting [40].

First, Galactose (Gal) decorated nanoparticles (NPs) were widely used to deliver drugs to cancer cells overexpressing asialoglycoprotein receptor (ASGR), such as liver cancer cell [41]. Building upon this, Anter et al. developed a nanoplatform called ‘Apocynin (APO)-loaded galactosylated chitosan(GC)-coated poly(d,l-propylene-ethylene-coated) nanoparticles’ (APO-loaded GC-coated PLGA NP). They achieved this by covalently coupling the galactose ligand, GC, with Apocynin (APO) for hepatocyte adhesion. The experimental results demonstrated that the system exhibited excellent hepatocyte targeting activity and the highest anti-cancer effect on the HepG2 cell line [42]. Second, as a biodegradable, biocompatible, and nontoxic disaccharide, lactic acid (LA) potentially can be used as an active targeting ligand for drug-loaded nanoparticles [43]. Cheng and his colleagues formed LA/CAT-DOX NPs by covalently concatenating the active targeting ligand LA and the chemotherapeutic drug DOX with catalase (CAT) via an EDC concatenation reaction. These nanoparticles were then co-assembled with the photosensitizer dihydroporphyrinol e6 (Ce6) via a straightforward mixing procedure to form LA/CAT-DOX-Ce6 nanoparticles. This hybrid nano-enzymatic drug delivery system containing the cytotoxic drug doxorubicin (DOX) can alleviate hypoxia in the tumor cell microenvironment and enhance chemotherapeutic sensitivity [44]. In addition to mono- and disaccharide targeting ligands, polysaccharide targeting ligands like hyaluronic acid (HA) and nanomaterials coupled and synthesized to target HA-based drug nanoparticles have shown important effects in improving drug delivery to cancer cells [45]. Hyaluronic acid can be conjugated to nanocarriers in two different ways: covalent binding and non-covalent binding (e.g., electrostatic interaction). The use of HA-based nanocarriers synthesized in different ways for the treatment of tumors with increased expression of CD44 receptor has been shown to be useful for improved drug delivery, increased cytotoxicity, and significant tumor growth inhibition. Furthermore, it has high potential for targeted chemotherapy [46,47].

Carbohydrate-based nanocarriers provide a new pathway for targeted delivery of anticancer drugs to tumors. With high specificity and multiple drug delivery capabilities, they can improve delivery by improving solubility, prolonging circulation time, and allowing the employed therapeutic agents to penetrate deeper into the tumor, and are promising tools for achieving selective drug delivery to target cells after glycan-drug coupling agent-mediated targeting [8]. However, the study of carbohydrate -modified nanoparticles is in the preliminary stage and there are a large number of problems to be solved, but it is believed that with the development of technology, carbohydrate -modified NPs can make more progress.

## 4. Peptide-Mediated Targeted Drug Delivery System for Tumor Treatment

The peptide is a low molecular weight ligand composed of fewer than 50 amino acids [48]. Peptides can bind specifically to receptors expressed on the cell surface, inside the cell or in the extracellular matrix with high affinity, making them a very good targeting ligand. Compared with antibodies or proteins, peptides are smaller in size (between small molecules and antibodies) and have a greater ability to penetrate cells or tissues. Their pharmacokinetics can be enhanced through chemical modifications, while their targeting ability remains largely unaffected [49]. Currently, drug delivery systems based on two peptide ligands, tumor targeting peptide and cell penetrating peptide, have been widely developed for cancer therapy. These systems not only alleviate the systemic side effects caused by chemotherapeutic agents but also significantly enhance therapeutic efficacy, delivery, and cancer targeting (Table 2). This sub-section reviews two main peptide-targeted drug delivery systems for cancer therapy applications: peptide-drug conjugates and peptide-conjugated nanoparticles [50,51].

### 4.1. Peptide-Drug Conjugates

In recent years, peptide-drug conjugates (PDCs) have gained attention as a promising area of research in cancer therapy. A complete PDC consists of a peptide, a linker, and a payload that covalently binds the peptide molecule to the small molecule drug through the linker [64].

To be effective, PDCs need to meet the condition of not releasing the drug prematurely during circulation and only releasing it at the tumor site. The choice of linker is crucial, as it not only ensures the stability of the PDC during circulation but also enhances the efficiency of cytotoxic drugs in killing tumor cells [65]. Linkers can be classified into cleavable and non-cleavable categories based on their stability in the body and the mechanism of cleavage at the tumor tissue. Cleavable linkers can further be divided into three types: enzyme-sensitive, acid-sensitive, and reduction-sensitive linkers [9]. For instance, Liu et al. proposed a novel doxorubicin peptide-drug conjugate (DOX PDC) that utilized a homodimeric HER-2 targeting peptide covalently conjugated with an acid-sensitive hydrazone bond to enhance tumor targeting ability and anticancer activity. Both in vitro and in vivo experiments demonstrated that this PDC effectively delivered DOX into HER2-positive SKBR-3 cells, significantly improving anticancer efficacy and reducing the side effects of DOX [66]. This research provides a new targeted delivery strategy for developing stable PDCs for anticancer therapy. In contrast, non-cleavable linkers such as thioethers, oximes, and triazoles do not undergo cleavage. These linkers rely on lysosomal/endosomal degradation after internalization of the drug conjugate to activate the drug. For instance, Yu et al. connected a cell-permeable peptide (Kip-related protein, (KRP)) with doxorubicin hydrochloride (DOX) through sulfide and amide bonds, resulting in a KRP-DOX conjugating. This conjugating was intravenously injected into mice with osteosarcoma. The study found that there was minimal release of free DOX in the bloodstream after intravenous injection, indicating good tumor tissue selectivity and tumor cell internalization efficiency. This was attributed to the stable covalent bond in the conjugating, which prevented premature drug release in the blood. Most of the DOX entered the tumor cells through KRP [67]. In preclinical trials, peptide drug conjugates usually show good biological activity and drug stability, but in the actual clinical setting, PDCs will also face some challenges, for example, the heterogeneity of tumor tissues will affect the targeting effect of PDCs, the existence of multiple metabolic enzymes and clearance mechanisms in the human body may lead to the degradation or rapid clearance of PDCs, and in addition, PDCs may cause an immune response in the body or produce toxic side effects. Thus, to date, there have been only 96 clinical trials of PDCs targeting antigens overexpressed in solid tumors. PDC clinical trials are only in phases I and II, which focus on the safety and efficacy of the drug in patients [68]. Nonetheless, there have been some successes, such as in 2021, when the FDA approved the first PDC, Pepaxto, for the treatment of relapsed or refractory multiple myeloma [69].

Compared with ADCs or other macromolecular polymeric drugs, PDC drugs have the advantage of smaller molecular size, stronger tumor tissue penetration ability, and enhanced permeability and retention effect [70]. However, the poor intrinsic pharmacokinetic properties of peptides raise concerns about long-term safety and efficacy. Furthermore, the development of new PDC modalities such as cyclotoxin conjugates, self-assembled PDCs, etc. should be supported in the near future using new technologies. The field of these drug conjugates continues to advance as promising drug delivery systems for cancer treatment [50].

### 4.2. Peptide-Conjugated Nanoparticles

In recent years, peptide targeting ligands have been increasingly important in drug delivery systems for nanomedicine applications. Self-assembled peptides or peptide-nanomaterials show great potential because of their low toxicity and remarkable therapeutic efficacy [71].

Currently, there are two main methods of conjugating peptides to nanomaterial delivery systems: covalent interactions and non-covalent interactions. Covalent interactions involve chemical linkage, where peptides are conjugated to nanocarriers through chemical bonds like ester bonds and amide bonds. For instance, Hao et al. prepared a GSH-responsive prodrug (PTX-SS-HPPH) by introducing a disulfide bond between paclitaxel (PTX) and photosensitizer 2-(1-hexyloxyethyl)-2-devinyl pyropheophorbide-a (HPPH), and then synthesized PTX-SS-HPPH /Pt@RGD-NP by modifying PTX-SS-HPPH and PtNP precursors with distearoyl phosphatidylethanolamine-polyethyleneglycol-RGD peptide (DSPE-PEG-RGD) via EDC/NHS chemistry. This modification enhanced the tumor targeting ability and permeability of the precursors and improved the photodynamic therapeutic efficiency of photodynamic therapy for bladder cancer [59]. On the other hand, non-covalent interactions involve methods such as electrostatic adsorption of opposite charges and hydrophobic interactions to form self-assembled peptide nanoparticles [72]. For example, Jiang et al. designed a multifunctional peptide (P51) for programmed delivery of the hydrophobic chemotherapeutic drug pyroxorubicin. First, these peptides act as a linker between negatively charged sequences and 41-residue peptides containing α-helices, which can self-assemble into stable spherical nanoparticles (P51-THP NPs) by entrapping pyroxorubicin through electrostatic and hydrophobic interactions. They have more effective tumor targeting, antitumor effects and reduced systemic toxicity [73]. Chen et al. co-loaded dabrafenib (Da) and doxorubicin (Dox) onto a self-assembled peptide nanofiber (Biotin-G^D^F^D^F^D^YGRGD, termed SPNs) via non-covalent interactions to form supramolecular self-assembled peptide nanofibers (SPNs/Da/Dox) for targeted and synergistic treatment of thyroid cancer. The experimental results showed that encapsulation in SPNs significantly enhanced the killing ability of Da and Dox, and SPN/Da/Dox showed targeted killing of cells with high BRAF V600E expression [74].

The use of peptide-modified nanoparticles for delivering drugs to cancer cells has gained increasing interest. These peptide nanomaterial delivery systems utilize the EPR effect to deliver prodrugs to targeted tumor tissues, resulting in higher aggregation at the tumor site and more efficient intracellular uptake compared with a single polymer chain, thus improving the effectiveness of tumor therapy. It also solves many problems in current nanoparticle-based drug delivery systems, including low drug loading efficiency, inherent nanoparticle toxicity, and limited targeting efficiency [75]. With the deepening research on active targeting materials and nanocarriers, it is reasonable to believe that in the near future, peptide-mediated targeted drug nanopreparations will enter the stage of clinical application, which will provide preparation guarantee for precise delivery of chemotherapeutic drugs and molecularly targeted drugs for tumor therapy.

## 5. Aptamer-Mediated Targeted Drug Delivery System for Tumor Therapy

**Figure 4:**
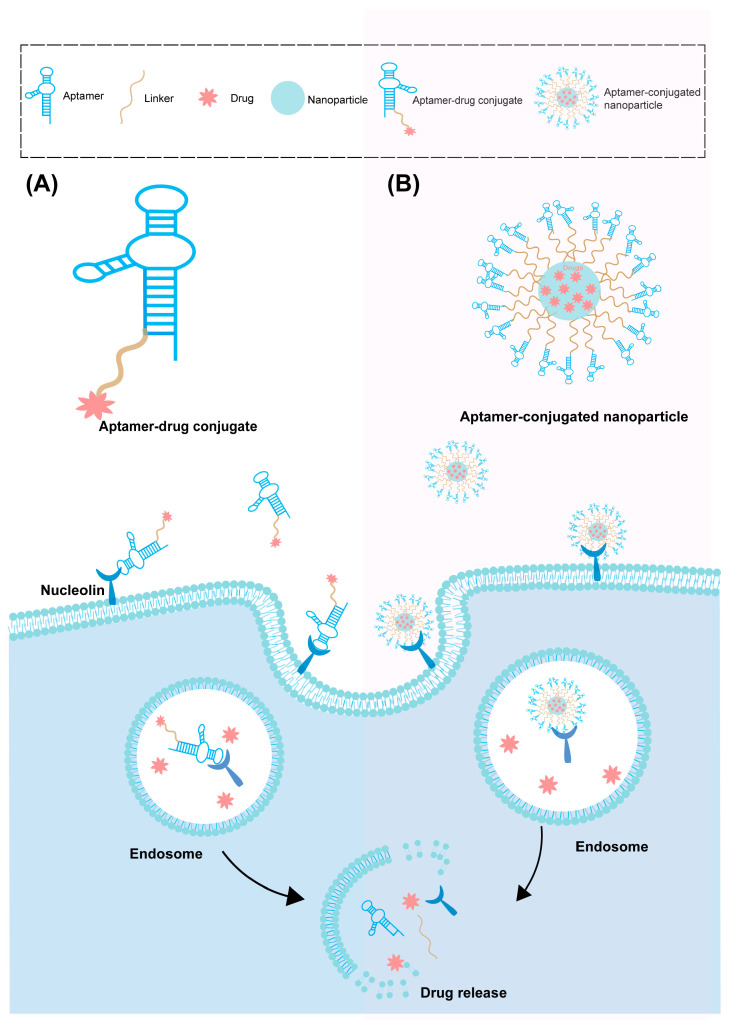
Aptamer-mediated targeted drug delivery system. (**A**) Targeting process of aptamer-drug conjugates. (**B**) Targeting process of aptamer conjugated nanoparticles.

Nucleic acid aptamers, which are short single-stranded DNA (ssDNA) or RNA oligonucleotides, possess specific secondary and tertiary structures. These aptamers are generated through systematic evolutionary screening for exponential enrichment of targeting ligands (SELEX) [76]. Nucleic acid aptamers are called chemical antibodies, but with specificity and affinity equal to, or better than, antibodies. Compared with traditional small molecule targeting ligands, aptamers offer advantages such as easy synthesis, facile chemical modification, good repeatability, high stability, and high specificity towards cell surface aptamer targets. These features make them useful in various applications, including biosensors, nanosystems (such as fluorescent/electrochemical probes and drug delivery vehicles), cancer diagnosis, and therapy. By enhancing receptor reawakening and cellular uptake, they contribute to improving therapeutic efficacy [77,78]. This subsection focuses on recent advances and challenges in aptamer-mediated targeted drug delivery systems in cancer therapy. It specifically discusses two main categories: aptamer-drug conjugates (ApDCs) and aptame-conjugated nanoparticles [79,80] (Figure 4).

### 5.1. Aptamer-Drug Conjugates

Aptamers have proven to be highly effective as small molecule delivery platforms in cancer therapy. Similar to antibody drug conjugates, aptamer drug conjugates (ApDCs) consist of three main components: aptamer, linker, and small molecule drug (often referred to as payload). The conjugating of nucleic acid aptamers and small molecule drugs can occur through covalent conjugating or physical interaction [81].

Covalent conjugating involves the formation of a covalent bond between the nucleic acid aptamer and the drug by modifying the reactive group, such as amino, sulfhydryl, or cyclooctyl. Zhang et al. developed UM (uveal melanoma) targeting ApDC by coupling the XQ-2d aptamer with the small molecule monomethyl aurisatin E (MMAE). This aptamer-drug coupling allowed specific binding of XQ-2d to UM cells through CD71 targeting. The results demonstrated significant UM targeting and anti-proliferative activity against UM both in vitro and in vivo, suggesting the potential of XQ-2dMMAE as a novel anti-tumor drug for UM treatment [82]. Physical interactions, on the other hand, were an early and widely used method for constructing aptamer-drug conjugates. Aptamers can bind non-covalently to small molecule drugs through electrostatic interactions, hydrophobic interactions, and other mechanisms. Henri et al. combined the EpCAM aptamer with Adriamycin (DOX) through hydrophobic interactions to form an aptamer-drug conjugate. This ApDC specifically binds to EpCAM proteins on the cell membrane of ovarian cancer cells and is internalized into the lysosome. Within the acidic environment of the lysosome, the ApDC releases Dox, leading to tumor cell death. The results demonstrated similar cytotoxicity in reducing tumorigenicity as DOX release, with reduced side effects due to the targeted nature of drug delivery [83]. In addition to targeting specific biomarkers, aptamers can also be used as therapeutics to modulate their biological function. Pegaptanib, the first therapeutic aptamer approved by the US FDA, is a polyethyleneglycolated anti-VEGF aptamer used for the treatment of age-related macular degeneration (AMD) [84]. The DNA aptamer AS1411 can work as a targeting ligand and therapeutic agent. It has shown the capacity to inhibit tumor cell development in a range of cancer cell lines and was applied in phase 1/2 clinical trials at the beginning of the 21st century [85].

Aptamer-drug conjugates and therapeutic aptamers have very promising clinical applications because of their excellent efficacy in cancer treatment. Although many ApDCs have been reported by researchers, few of them have made it to clinical trials. This is due to the low in vivo specificity, low serum stability and rapid renal clearance of ApDCs. Therefore, the development of ApDCs is in its infancy and there is a long way to go before clinical translation.

### 5.2. Aptamer-Conjugated Nanoparticles

Aptamers, known for their high affinity and specificity, can be easily conjugated to the chemical group ends of nanoparticles. This conjugation does not significantly increase the size of the nanoparticles, while enhancing their drug loading capacity compared with chemically conjugated targeted drugs. Consequently, aptamer-functionalized nanocarriers serve as intelligent drug carriers with remarkable drug delivery and targeting properties [86]. The integration of aptamers and nanotechnology has facilitated the development of various targeted drug delivery systems for clinical therapy and diagnostics.

Two primary strategies have been reported for efficiently incorporating aptamers into nanocarriers: physical encapsulation via electrostatic interactions and chemical conjugation via covalent bonds [87]. Physical encapsulation, utilizing electrostatic interactions, is the most commonly employed strategy due to the negative charges exhibited by aptamers [88]. For instance, Darabi et al. designed and synthesized solid lipid nanoparticles (SLN/DOX/Dexa) with positive charges, incorporating adriamycin (DOX) and dexamethasone (Dexa). These nanoparticles were then bound to negatively charged anti-EGFR/CD44 dual RNA aptamers through electrostatic interactions, resulting in SLN/DOX/Dexa/CD44/EGFR nanoparticles. Experimental results demonstrated that these nanoparticles effectively inhibited the proliferation of triple-negative breast cancer cells and improved tumor therapy efficiency. This study suggests that the dual targeting of DOX-SLN using two nucleic acid aptamers holds promise as a combination therapy [89]. Another strategy for binding nucleic acid aptamers to nanomaterials is through covalent binding. Torabi et al. conducted a study where they loaded sunitinib onto magnetic mesoporous silica nanoparticles and covalently coupled them with MUC-1 aptamers. This novel approach aimed to develop a targeted delivery system for ovarian cancer cells that overexpress MUC-1 glycoprotein. The experimental results demonstrated that this aptamer-oriented targeting nanosystem specifically targeted advanced ovarian cancer cells. Consequently, it enhanced the uptake of anticancer drugs by tumor cells, overcame drug resistance, and significantly improved the efficiency of tumor treatment [90].

In preclinical experiments, aptamer-conjugated nanoparticles have demonstrated encouraging outcomes for tumor therapy. These nanoparticles are able to bind to targets on the surface of tumor cells via specific aptamers for precise drug delivery. However, there is a lack of drugs with aptamer-functionalized nanoparticles on the market. This is mainly due to the fact that there are some barriers to developing aptamer-coupled nanoparticles for clinical applications. The selection of suitable aptamers as targeting ligands is critical. In addition, future efforts need to focus on aptamer screening and biotechnology optimization to further improve the efficacy and safety of aptamer- conjugated nanoparticles. Targeted therapies based on aptamers are expected to prolong survival times for cancer patients and reduce drug resistance. Therefore, further research and development in this area is of great clinical importance.

## 6. Antibody-Mediated Targeted Drug Delivery System for Tumor Therapy

**Figure 5:**
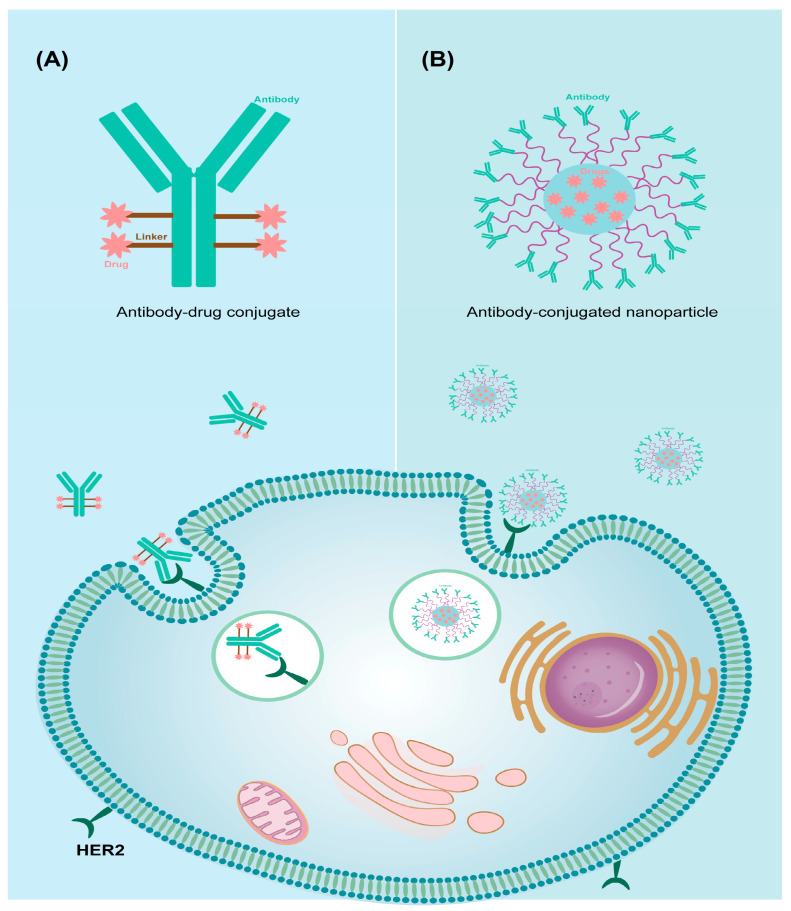
Antibody-mediated targeted drug delivery system. The system is divided into two main strategies: (**A**) Antibody-drug conjugates. (**B**) Antibody-conjugated nanoparticles.

Monoclonal antibodies (mAbs) have been successfully utilized in experimental and clinical settings to target cancer-specific antigens, playing a crucial role in modern cancer therapy [91]. In the 1970s, chemotherapy based on monoclonal antibodies (mAbs) became available. Currently, the US Food and Drug Administration (FDA) has approved approximately 30 antibodies for the direct treatment of cancer, rheumatoid arthritis, Crohn’s disease, and antiviral prophylaxis [92]. However, cancer treatment extends beyond a single drug, as different drugs can synergistically act together. By combining antibodies with chemotherapeutic agents, improved therapeutic outcomes can be achieved. Moreover, antibodies are also employed as targeting ligands for drug delivery systems. Their high specificity enables them to selectively deliver drugs to cancer cells, minimizing damage to normal tissue. In this review, we focus on two major strategies that are currently being investigated or have received clinical approval for combining chemotherapeutics with antibodies: antibody-drug conjugates (ADCs) and antibody-conjugated nanoparticles [93,94] (Figure 5).

### 6.1. Antibody-Drug Conjugates

Antibody-drug conjugates (ADCs) are emerging novel anticancer drugs consisting of three components: a tumor-specific antibody or antibody fragment, a cleavable or non-cleavable chemical conjugate and a potent cytotoxic molecule. Due to the targeting advantages of monoclonal antibodies and the cytotoxicity of small molecule drugs, ADCs are emerging as a new cancer treatment option [95,96].

Two main types of conjugation are commonly used in ADC design: conventional conjugations and site-specific conjugations [97]. For a long time, the conventional approach to ADCs has used lysine or cysteine residues that are exposed on the surface as anchoring sites to join drug molecules. This choice is based on the fact that thiol groups are widely present in living organisms and have a high capacity to interact with other biomolecules such as proteins and enzymes. For instance, Ado-trastuzumab emtansine (T-DM1) is one of the four approved ADCs on the market that utilizes side-chain lysines to link the potent microtubule protein inhibitor DM1 to the HER2 antibody trastuzumab for the treatment of HER2-positive metastatic breast cancer [98]. While this approach is easy to apply, these conventional conjugation methods result in multiphase byproducts with different drug distributions for each mAb, unspliced and overspliced mAbs. In contrast, site-specific concatenation using genetically engineered sites is an effective method for achieving more homogeneous ADCs. This method mainly links medications and antibodies particularly by employing glycans, short peptide tags, unnatural amino acids, or specific amino acids [99]. For instance, the homogenous anti-HER2 ADC ARX788, which was created in 2020, produces a drug-to-antibody ratio of 1.9 by means of a non-removable abelastatin (AS269) drug junction and a special unnatural amino acid affixation technique. The study’s findings demonstrated that ARX788 outperformed T-DM1 in xenograft models with HER2 overexpression and HER2 deficiency, and it effectively suppressed tumor growth. Furthermore, ARX788 showed notable anti-tumor effect against HER2-positive and HER2 low-overexpressing tumors, as well as efficacy in T-DM1-resistant models, in xenograft experiments conducted on patients with breast and gastric malignancies [100].

ADCs are widely used in tumor therapy, and to date, the FDA has approved 14 ADCs as single or combination agents for clinical use in the treatment of various types of cancer. For example, Trastuzumab deruxtecan [101], Tivdak^TM^ (tisotumab vedotin-tftv) [102] and mirvetuximab soravtansine (ELAHERE™) [103], etc. Meanwhile, there are more than 150 ADCs that are in various stages of clinical trials for the treatment of various types of cancer alone and in combination with other chemotherapeutic agents and have shown good results [104]. While there have been promising outcomes with antibody-drug couplers in clinical trials, the application of ADCs in the clinical setting presents certain disparities and challenges compared with preclinical trials. One such challenge is the limited ability of ADCs to penetrate deeply into solid tumors due to the large size of the antibodies. And the human immune system may generate an immune response to ADCs, resulting in their degradation and clearance in vivo [105]. Additionally, the extended circulation cycle of ADCs can lead to premature drug release and potential adverse effects on normal tissues within the body [106].

The development of ADCs has brought significant therapeutic benefits to cancer patients. Meanwhile, for the future prospect of ADC, it is believed that it can continue to move forward by further changing the conjugating technology of ADC toward the direction of directed coupling technology and synthesizing multivalent conjugated ADC drugs.

### 6.2. Antibody-Conjugated Nanoparticles

Antibody-conjugated nanoparticles are a promising medical platform for targeted drug delivery [107]. There are two main strategies for synthesizing antibody-nanoparticle conjugates: physical adsorption and covalent conjugation [108].

Physisorption, the first strategy, is a simple non-covalent immobilization method that relies on hydrogen bonding, Van der Waals forces, hydrophobic, and electrostatic interactions. This method does not require any chemical modification of the antibody or nanoparticle; instead, they are mixed together to attach the antibodies to the nanoparticle surface [109]. For instance, Li et al. demonstrated the self-assembly of ce6-conjugated hyaluronic acid (HC), dextro-1-methyl tryptophan-conjugated polylysine (PM), and aPDL1 into aPD-L1@HC/PM NPs through electrostatic adsorption. These nanoparticles enabled tumor immunotherapy at the all-immune stage [110]. On the other hand, covalent coupling is the most common approach for developing antibody-nanoparticle conjugates. It involves techniques such as carbodiimide chemistry, maleimide binding, and click chemistry [111]. Compared with adsorption methods, covalent strategies provide stable and reproducible antibody-nanoparticle couplings. For example, S. Jain et al. utilized EDC/NHS chemistry and DSPE-PEG-COOH as a linker to conjugate VEGF antibodies to pH-sensitive DTX liposomes, resulting in VEGF antibody functionalized PEGylated pH-sensitive liposomes (VEGF-PEG-pH-Lipo-DTX). This development enhanced the therapeutic effectiveness of DTX while reducing associated side effects [112].

On the market currently, there are many types of non-functionalized nanoparticles that can be used to treat cancer. Antibody-functionalized nanoparticles, however, have a limited number of studies, with most studies at the formulation, in vitro, and preclinical investigation stages, including animal models based on xenografts. Only a few studies have progressed to clinical trials, specifically phase 1 and 2 trials [113]. For instance, in one open-label, phase 1 clinical study, doxorubicin-loaded immunoliposomes targeting cetuximab Fab fragments were evaluated for their safety, pharmacokinetics, and efficacy. The study found that the anti-EGFR immunoliposomes were well-tolerated at lower doses (up to 50 mg doxorubicin per m²), with most adverse events attributed to tumor progression [114]. Currently, a phase 2 clinical trial is recruiting patients with advanced triple-negative breast cancer to evaluate the effectiveness of doxorubicin-loaded anti-EGFR immunoliposomes [115].

Antibody-nanoparticle conjugate systems have shown promise in enhancing tumor targeting of therapeutic agents and minimizing toxic side effects, making them highly regarded in cancer diagnosis and therapy. However, several fundamental issues related to the preparation of these conjugates remain unresolved. These include understanding the impact of linker length on cellular uptake, biodistribution, metabolism, and long-term toxicity of nanoparticles. Thus, future research should focus on selecting suitable modification and conjugation strategies and reagents to achieve even more efficient tumor targeting.

## 7. Conclusions and Future Perspectives

With the increasing incidence of cancer, there has been a rise in research on cancer treatments. However, the drug resistance of cancer cells, along with recurrence and metastasis, presents challenges in eradicating cancer. The primary objective of cancer treatment is to accurately target and eliminate cancer cells. Currently, small molecule chemotherapeutic drugs and nanomedicines are widely used as cancer therapeutic methods. However, these drugs often lack the ability to specifically target tumors, leading to high toxicity and side effects on normal cells and tissues. Therefore, it is necessary to modify these drugs to enhance the efficiency of cancer treatment [5]. Targeting ligands, which are drug carriers that selectively target tumor cells, have effectively addressed these drug-related limitations. By modifying small molecule chemotherapeutic drugs and nanomedicines, a targeted drug delivery system can be created, enabling precise localization of tumor cells and controlled drug release. This improves the drug’s effectiveness and reduces toxic side effects.

In this review, we provide a summary of recent research progress on various targeted ligand-mediated drug delivery systems, each with its unique advantages and corresponding limitations. However, there are many problems associated with targeted drug delivery systems, such as decreased targeting due to the selection of inappropriate targeting ligands, low therapeutic efficacy of drugs even when they reach the target (cancer cells) due to the formation of protein corona on the surface of the targeted delivery system, and toxic side effects, as well as the complexity and inefficiency of the targeted drug delivery system and the clinical translational efficiency. Therefore, future research on targeted drug delivery systems needs to continuously explore new targeting ligands through molecular biology methods, bioinformatics tools, and molecular engineering techniques to improve targeting specificity based on ensuring targeting and biocompatibility. The interference of the protein corona should also be minimized by surface modification of the nanocarriers, density control of the targeting ligands, and the use of “invisible” materials (e.g., polyethylene glycol (PEG)). Furthermore, simplifying the system design, conducting comprehensive safety and efficacy assessments, and promoting interdisciplinary collaboration are crucial for balancing the complexity of the targeted delivery system and facilitating its translation from the laboratory to the clinic. This will ultimately enhance therapeutic efficacy and ensure patient safety.

## Figures and Tables

**Table 1:** Different types of targeting ligands: a summary of their structures, advantages and disadvantages.

Targeting Ligands	Structure	Advantages	Disadvantages
Folic acid	Water-soluble vitamin composed of 3 ingredients: pteridine, p-aminobenzoic acid and glutamic acid.	Small size, chemical simplicity, biocompatibility and low cost	Limited aiming, limited load capacity
Carbohydrates	Organic compounds composed of the elements carbon, hydrogen and oxygen	Naturally occurring, biocompatible, structural diversity	Structural complexity, targeting and affinity variation
Peptides	Compounds formed by the dehydration and condensation of 10–100 amino acid molecules	Highly customizable, good biodegradability	Poor stability, high preparation cost, hhort half-life
Aptamers	A short sequence of oligonucleotides or a short polypeptide obtained by in vitro screening	High specificity and affinity, easy to synthesize, easy for chemical modification, good reproducibility	Lower immunogenicity, metabolic instability
Antibodies	A class of immunoglobulins that bind specifically to antigens	High purity, high sensitivity, high specificity, low cross-reactivity, high immunogenicity	High preparation cost, difficulty in chemical modification

**Table 2:** Peptide-targeted drug delivery systems.

Type	Peptide	Peptide Name	Drug	Cancer Model	References
Peptide-drug conjugates	TTP	EDB-FN targeted peptides	Doce and Dox	Prostate cancer	[52]
		breast cancer cell targeting peptide	Dox	Breast cancer	[53]
		GPC3-targeting peptide	Ce6	Hepatocellular Carcinoma	[54]
	CPP	TAT peptide	PTX	Brain glioma	[55]
		LMW peptide and TAT peptide	PTX	Lung cancer	[56]
		T2 peptide	PTX	Breast cancer	[57]
Peptide-conjugated nanoparticles	TTP	P1c peptide	DOX	Glioblastoma	[58]
		RGD peptide	PTX and HPPH	Bladder cancer	[59]
		AR peptide	ICG and DOX	Breast cancer	[60]
	CPP	TAT peptide	PTX	Lung cancer	[61]
		TAT peptide	PTX	Breast cancer	[62]
		Sv peptide	Gold Nanoclusters	Refractory lymphomas	[63]

Abbreviations are as follows: (TTP): tumor targeting peptide, and (CPP): cell penetrating peptide.

## Data Availability

The data presented in this study are available in this article.
